# Supplementary material for: Mapping the evolution of fertility support policies in China: A content and instrumental analysis
Source: PLoS One. 2025 Oct 9;20(10):e0332137. doi: 10.1371/journal.pone.0332137 (PMC12510515; doi:10.1371/journal.pone.0332137)
Supplement: S1 Appendix — (ZIP) [file pone.0332137.s001.zip › S1 Appendix. 226 original policy documents/43-国家卫生健康委关于做好托育机构相关工作的通知(FBM-CLI-4-338907).doc]

国家卫生健康委关于做好托育机构相关工作的通知

**发布部门：** [国家卫生健康委员会](https://www.pkulaw.com/law/chl?Aggs.IssueDepartment=60321) [机构沿革](javascript:void(0))

**发文字号：**国卫人口函〔2020〕23号

**发布日期：**2020.01.28

**实施日期：**2020.01.28

**时效性：** [现行有效](https://www.pkulaw.com/law/chl?Aggs.TimelinessDic=01)

**效力级别：** [部门规范性文件](https://www.pkulaw.com/law/chl?Aggs.EffectivenessDic=XE0302)

**法规类别：** [公共场所与环境卫生](https://www.pkulaw.com/law/chl?Aggs.Category=09619) [卫生防疫检疫](https://www.pkulaw.com/law/chl?Aggs.Category=09616) [疫情防控](https://www.pkulaw.com/law/chl?Aggs.Category=122)

**专题分类：** [突发事件](https://www.pkulaw.com/law/chl?Aggs.SpecialType=005)

国家卫生健康委关于做好托育机构相关工作的通知
（国卫人口函〔2020〕23号）

各省、自治区、直辖市及新疆生产建设兵团卫生健康委：
　　根据党中央、国务院关于新型冠状病毒感染的肺炎疫情防控工作的总体部署，落实《[国务院办公厅关于延长2020年春节假期的通知](https://www.pkulaw.com/chl/2f5c97ef07fa381abdfb.html)》（国办发明电〔2020〕1号），切实保障3岁以下婴幼儿生命安全和身体健康，现就做好托育机构有关工作通知如下：
　　一、各地卫生健康行政部门要按照地方党委、政府的统一部署，切实发挥牵头作用，积极协调相关部门按照各自职责，加强对托育机构的监督管理。

　　二、指导各类托育机构可依法暂停开展收托、保育服务，具体恢复入托时间根据实际情况确定。

　　三、指导面向3岁以下婴幼儿的早教机构、亲子园等，暂停开展线下培训活动，鼓励利用互联网等信息化手段提供服务。
　　工作中遇到的困难和问题，及时向我委报告。
　　联系人：人口家庭司　梁效革、徐拥军
　　联系电话：010-62030572、62030585
　　传　　真：010-62030790

　　国家卫生健康委
2020年1月28日

© 北大法宝：（[www.pkulaw.com](https://www.pkulaw.com/)) 专业提供法律信息、法学知识和法律软件领域各类解决方案。北大法宝为您提供丰富的参考资料，正式引用法规条文时请与标准文本核对。
欢迎查看所有[产品和服务](https://www.pkulaw.com/helps/2.html)。法宝快讯：[如何快速找到您需要的检索结果？](https://www.pkulaw.com/helps/70.html)[法宝](https://www.pkulaw.com/helps/69.html)V6有何新特色？

扫描二维码阅读原文

**原文链接：**<https://www.pkulaw.com/chl/780336f0e8f0a656bdfb.html>
